# Factors associated with hospitalization of people with influenza in a Malaysian tertiary hospital from 2015 to 2019

**DOI:** 10.1371/journal.pone.0333921

**Published:** 2025-10-17

**Authors:** Rakhee Yadav Hematram Yadav, Nithiah Thangiah, Karumathil Sharmila Bhaskaran, Chitra Kumarasamy, Shathanapriya Ammikapathi, Jean Khor, Sanjay Rampal

**Affiliations:** 1 Ara Damansara Medical Centre Member of Asia OneHealthcare, Shah Alam, Selangor, Malaysia; 2 Centre for Population Health, Department of Social and Preventive Medicine, Faculty of Medicine, Universiti Malaya, Kuala Lumpur, Malaysia; 3 Centre of Epidemiology and Evidence-Based Practice, Department of Social and Preventive Medicine, Faculty of Medicine, Universiti Malaya, Kuala Lumpur, Malaysia; 4 Sanofi (Malaysia), Petaling Jaya, Selangor, Malaysia; Aga Khan University, PAKISTAN

## Abstract

Influenza contributes substantially to global morbidity and mortality. While most individuals recover from influenza without complications, certain groups are at higher risk of developing severe illness requiring hospitalization. This retrospective cohort study investigated the plausible determinants of hospitalization and its duration among participants who tested positive for influenza A or B. A hospital-based study was conducted in Selangor, Malaysia from 1 January 2015 until 31 December 2019. Sociodemographic characteristics, laboratory investigations, clinical signs and symptoms, and comorbidities were extracted from the hospital’s electronic medical records. Binary logistic and multiple linear regression were used to model the odds of hospitalization and duration of stay, respectively, stratified by age (<18 and ≥ 18 years). A total of 2,593 participants comprising 1,420 < 18 and 1,173 ≥ 18 years were included. Among <18, the odds of hospitalization were higher among those aged under 5 compared to 5–17 years, Malays compared to Chinese, and those with fever, cough, and chest x-ray. Hospitalization duration was higher among Malays and Indians compared to Chinese children, and those with fever, cough and chest x-ray. Among adults, those aged 18–39 years compared to 40–59 years, Malays and Indians compared to Chinese, those with a history of fever, cough, sore throat and chest x-ray were more likely to be hospitalized. The duration among ≥ 18 years was higher among those ≥60 years, chest x-ray, and females. Participants under 5 years are more likely to be hospitalized and those aged 60 and above were more likely to have longer hospital duration compared to all other ages and may benefit more from primary prevention activities such as vaccination. The heterogeneous associations between the risk factors for hospitalization and hospital duration highlight the need for further research. Better risk stratification of influenza patients may better mitigate the impact of influenza A and B.

## Introduction

Influenza is a significant contributor to major respiratory illness and mortality worldwide, with annual cases affecting 5–10% of adults and 20–30% of children [1]. This results in 3–5 million cases of severe illness and 290,000–650,000 cases of respiratory deaths worldwide [2,3]. South-East Asia is reported to have the second-highest influenza-specific mortality rates ranging from 3.5 to 9.2 per 100,000 individuals [2]. The majority of reported deaths related to influenza occur in individuals aged 65 and above [4]. While most individuals recover from influenza without complications, certain groups, including children and the elderly are at higher risk of developing severe illness, requiring hospitalization [5–8].

The World Health Organization (WHO) strongly advocates the establishment of surveillance and epidemiological studies in developing countries to inform public health strategies [9]. The Malaysia Influenza Surveillance System (MISS) was initiated by the Ministry of Health (MOH) in 2004, which was updated in 2015 to the Malaysia Influenza Surveillance Protocol (MISP), comprising 15 MOH health clinics sentinel sites for Influenza Like Illness (ILI) surveillance and 9 MOH hospitals for Severe Acute Respiratory Infection (SARI) surveillance [10].

Influenza activity in tropical countries is complex and variable, and may not display the defined seasons seen in temperate countries. As a country close to the equator, Malaysia has a background of year‐round transmission with no clear seasonality [11,12]. Local studies indicate that influenza activity remains at low levels throughout the year, with peaks of seasonal transmission of varying duration occurring only in some years [13].

To our knowledge, there is no published report of influenza infections from a private healthcare facility in Malaysia. Published data on clinical features and health outcomes associated with influenza infection in Malaysia remains scarce. Therefore, we aimed to investigate the associations between sociodemographic characteristics, clinical signs and symptoms and co-morbidities with the risk of hospitalization and the ratio of its duration in laboratory‐confirmed people with influenza at a private hospital in the Klang Valley region of Peninsular Malaysia.

## Methods

The reporting of this study followed the STROBE guidelines for cohort studies.

This is a retrospective cohort hospital-based study in Selangor, Malaysia. The study population included <18 years and ≥ 18 years old participants who tested positive for influenza A or B based on the laboratory results. Clear guidelines on laboratory testing are followed based on institutional practice. Those <18 years are tested for influenza when they present or develop influenza-like illness (ILI) and severe acute respiratory infection (SARI). Participants ≥ 18 years with ILI symptoms are tested for influenza if their symptoms are severe and/or gave a significant history of contact with a person with influenza.

Influenza was diagnosed using a nasopharyngeal swab test. Samples were taken from various departments including the Emergency Department, Outpatient Clinics, wards and Critical Care Units that treat patients suspected with respiratory tract infection. Approximately 8,629 samples were tested for influenza A and B from 1 January 2015 until 31 December 2019. Influenza A and B were tested using Fujirebio (Immunochromatography) from 2015–2018 and SD Biosensor (Florescence Detection) from January 2019 to December 2019. Clinical sensitivity for Influenza A and Influenza B achieved 97% and 94.3% respectively whereas clinical specificity for both viruses was at 97.6% respectively when compared to real-time PCR tests.

Information on sociodemographic characteristics, laboratory investigations, clinical signs and symptoms, and pre-existing comorbidities were extracted from the hospital’s electronic medical records (EMR) in May 2021. This information included age, gender, ethnicity, history of fever (≥ 38.5°C), history of cough, history of sore throat, whether chest x-ray was taken before admission, and history of asthma, cardiovascular disease, diabetes, hypercholesterolemia, or hypertension. Each symptom was assessed at presentation (Yes/No). All participants information was entered on-site directly into a Microsoft Excel sheet. The data was reviewed and checked for completeness. All data was then verified against its original values in the EMR. Upon verification, no missing data were found.

**Fig 1:**
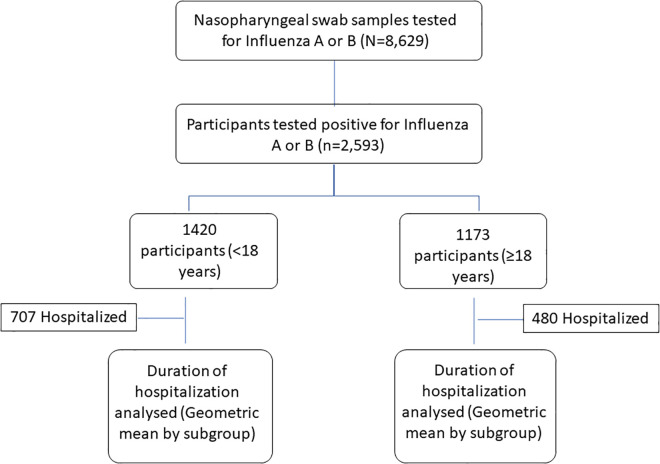
Participant flow diagram.

The two major clinical outcomes were hospitalization and its duration. Hospitalization of people with influenza was ascertained based on whether they were warded. The duration of hospitalization was estimated as the difference between the date of admission and the date of discharge. All analyses were stratified by age groups of <18 years and ≥18 years. Fig 1 shows the participant flow diagram. In order to reduce variability within groups, subgroup analyses were conducted within specific age groups to understand how the associations differ across ages. For the < 18 years, age groups were categorized as <5 and 5–17 whereas for the ≥ 18 years, age groups were categorized as 18–39, 40–59 and ≥60. These age categories reflect established risk groups for influenza while aligning with international guidelines and local surveillance practices [3,13,14].

### Statistical methods

Categorical variables are summarized using frequencies and percentages. Hospital duration is summarized as geometric means and 95% confidence intervals. Since the duration of hospitalization was positively skewed, the natural log of hospital duration was exponentiated and interpreted as geometric means. It is less sensitive to extreme values and is interpreted in the same units as the original data. The differences between proportions and means were statistically tested using Pearson’s chi-square test (or Fisher’s exact test for categorical variables with small cell counts) and Student’s t-test or F test, respectively. Due to the exploratory nature of this study, no adjustments were made for multiple comparisons to the Type I error, and this may result in a family wise Type I error higher than 0.05.

The odds of hospitalization were modelled using logistic regression. The natural log of hospital duration was modelled using linear regression since the duration of hospitalization had a skewed distribution. The exponentiated results are presented as hospitalization duration ratios.

Covariates considered in both logistic and linear regression models included sociodemographic, comorbid, and clinical characteristics. A stepwise forward selection model strategy was used to build the multivariable models with with an entry threshold of P < 0.20 [15]. The crude and adjusted odds and hospitalization duration ratios, their 95% confidence intervals, and the P values were reported. Crude estimates are calculated using univariate regression models while adjusted estimates are obtained from multivariate regression models. A two-sided Type I error was set at 0.05. The predictive performance and discrimination of the final multivariate logistic regression model was evaluated using the area under the receiver operating curve (AUC). The model diagnostics of the final multivariate linear regression included R-squared and F-statistic. All analysis was conducted in Stata version 14. The adjusted odds ratios and 95% confidence intervals for hospitalization were visualized using a forest plot to compare the magnitude of associations across predictors. The plot was generated using Microsoft Excel spreadsheet.

### Ethics statement

The study protocol was reviewed and approved by the Independent Ethics Committee, Subang Jaya Medical Centre with Institutional Review Board (IRB) reference number 202501.2. As all data were fully anonymized, no verbal or written consent was obtained.

## Results

From 8,629 nasopharyngeal swab samples, a total of 2,593 (n = 1,420 for <18 years and n = 1,173 for ≥18 years) participants who tested positive for influenza A or B were included in the analysis. The distribution of clinical outcomes is presented by sociodemographic and clinical characteristics for <18 years and ≥18 years in Tables 1 and 2, respectively.

**Table 1:** Hospitalization outcome and hospitalization duration in Influenza A or B participants aged < 18.

Characteristics	Overall (N = 1,420)	Hospitalization		P-value*	Hospitalization duration, Geometric Mean**	P-value
		No	Yes			
Age				<0.001		0.419
< 5 year	703	298 (42.4)	405 (57.6)		2.7 (2.6, 2.8)	
5–17 years	717	415 (57.9)	302 (42.1)		2.6 (2.5, 2.7)	
Gender				0.263		0.860
Female	642	333 (51.9)	309 (48.1)		2.7 (2.5, 2.8)	
Male	778	380 (48.8)	398 (51.2)		2.6 (2.5, 2.8)	
Ethnicity				<0.001		<0.001
Malay	921	392 (42.6)	529 (57.4)		2.7 (2.6, 2.8)	
Chinese	278	184 (66.2)	94 (33.8)		2.2 (1.9, 2.4)	
Indian	109	58 (53.2)	51 (46.8)		2.9 (2.5, 3.3)	
Others	112	79 (70.5)	33 (29.5)		2.9 (2.5, 3.4)	
History of:						
Fever				0.002		0.061
Yes	1,403	698 (49.8)	705 (50.3)		1.4 (0.0, 115.6)	
No	17	15 (88.2)	2 (11.8)		2.6 (2.6, 2.7)	
Cough				<0.001		0.003
Yes	1,247	598 (48.0)	649 (52.0)		2.7 (2.6, 2.9)	
No	173	115 (66.5)	58 (33.5)		2.2 (1.9, 2.6)	
Sore Throat				0.162		
Yes	481	229 (47.6)	252 (52.4)		2.6 (2.5, 2.7)	0.504
No	939	484 (51.5)	455 (48.5)		2.7 (2.5, 2.8)	
Chest X-ray				<0.001		<0.001
Yes	242	43 (17.8)	199 (82.2)		3.2 (3.0, 3.4)	
No	1,178	670 (56.9)	508 (43.1)		2.5 (2.4, 2.5)	
Asthma				0.193		0.567
Yes	49	20 (40.8)	29 (59.2)		2.5 (2.1, 3.1)	
No	1,371	693 (50.6)	678 (49.5)		2.6 (2.6, 2.7)	

Significant values: p < .05; *For each p-value presented, Fisher’s exact test of associations was used; **For each p-value presented, independent samples t-test was used except for ethnicity, analysis of variance was used.

**Table 2:** Hospitalization outcome and hospitalization duration in Influenza A or B participants aged ≥ 18.

Characteristics	Ov-1,173)	Hospitalization		P-value*	Hospitalization duration, Geometric Mean	P-value**
		No	Yes			
Age				0.462		0.121
18–39 years	707	411 (58.1)	296 (41.9)		2.6 (2.5, 2.8)	
40–59 years	333	206 (61.9)	127 (38.1)		2.5 (2.3, 2.7)	
≥ 60 years	133	76 (57.1)	57 (42.9)		3.1 (2.6, 3.7)	
Gender				0.635		0.086
Female	597	357 (59.8)	240 (40.2)		2.8 (2.6, 2.9)	
Male	576	336 (58.3)	240 (41.7)		2.6 (2.4, 2.7)	
Ethnicity				<0.001		0.923
Malay	596	315 (52.9)	281 (47.2)		2.7 (2.5, 2.8)	
Chinese	333	225 (67.6)	108 (32.4)		2.7 (2.4, 2.9)	
Indian	147	82 (55.9)	65 (44.2)		2.6 (2.3, 2.8)	
Others	97	71 (73.2)	26 (26.8)		2.6 (2.3, 3.0)	
History of:						
Fever				<0.001		0.445
Yes	1,124	650 (57.8)	474 (42.2)		2.6 (2.5, 2.8)	
No	49	43 (87.8)	6 (12.2)		3.1 (1.5, 6.4)	
Cough				<0.001		0.365
Yes	1,028	576 (56.1)	452 (44.0)		2.7 (2.6, 2.8)	
No	145	117 (80.7)	28 (19.3)		2.5 (2.0, 3.0)	
Sore Throat				<0.001		0.601
Yes	585	293 (50.1)	292 (49.9)		2.6 (2.5, 2.8)	
No	588	400 (68.0)	188 (32.0)		2.7 (2.5, 2.9)	
Chest X-ray				<0.001		0.004
Yes	279	55 (19.7)	224 (80.3)		2.8 (2.7, 3.0)	
No	894	638 (71.4)	256 (28.6)		2.5 (2.4, 2.6)	
Smoking				0.061		0.084
Yes	15	5 (33.3)	10 (66.7)		3.4 (1.9, 6.2)	
No	1,158	688 (59.4)	470 (40.6)		2.6 (2.5, 2.8)	
Asthma				0.030		0.214
Yes	33	13 (39.4)	20 (60.6)		3.0 (2.2, 4.2)	
No	1,140	680 (59.7)	460 (40.4)		2.6 (2.5, 2.8)	
Any comorbidity						
Cardiovascular diseases				0.405		0.167
Yes	5	2 (40.0)	3 (60.0)		1.8 (0.5, 7.2)	
No	1,168	691 (59.2)	477 (40.8)		2.6 (2.5, 2.7)	
Diabetes mellitus				0.085		0.064
Yes	36	16 (44.4)	20 (55.6)		3.2 (2.3, 4.5)	
No	1,137	677 (59.5)	460 (40.5)		2.6 (2.5, 2.7)	
Hypercholesterolemia				0.409		
Yes	1	0	1 (100.0)		2.7 (2.5, 2.8)	–
No	1,172	693 (59.1)	479 (40.9)		–	
Hypertension				0.002		0.028
Yes	71	29 (40.9)	42 (59.2)		3.1 (2.6, 3.7)	
No	1,102	664 (60.3)	438 (39.8)		2.6 (2.5, 2.7)	

Significant values: p < .05; *For each p-value presented, Fisher’s exact test of associations was used; **For each p-value presented, independent samples t-test was used except for age and ethnicity, analysis of variance was used.

The majority of people with influenza A or B were those < 5 years, 18–39 years, Malays and those presenting with fever, cough, sore throat and chest x-ray. The number ofpeople with influenza hospitalized was significantly higher among those < 5 years (57.6%) compared to those 5–17 years (42.9%) (Table 1). Similarly, the proportions were found to be higher among Malays (57.4%), followed by Indian (46.8%) and Chinese (33.8%) ethnic groups. A significantly higher proportion of <18 years hospitalized presented with certain clinical signs and symptoms compared to other partcipants. The majority of them either had a history of fever (50.3%), cough (52.0%) or needed a chest x-ray taken (82.2%). Significant differences in proportions were present between all groups (P < 0.001). Participants of the Indian ethnic group had a significantly longer length of stay with a geometric mean, GMreported at 2.9 (95% CI 2.5, 3.3) compared to other ethnic groups. Those with a history of cough (GM = 2.7; 95% CI 2.6, 2.9) and those who underwent a chest x-ray (GM = 3.2; 95% CI 3.0, 3.4) had significantly longer hospital stays on average compared to other partcicpants <18 years.

Similar to participants <18 years, the proportion of participants ≥18 years hospitalized due to influenza A or B was significantly higher among Malays (47.2%), followed by Indian (44.2%) and Chinese (32.4%) ethnic groups. A significantly higher proportion of those ≥18 years presenting with a history of fever (42.2%), cough (44.0%), sore throat (49.9%), hypertension (59.2%), and had a chest x-ray (80.3%) were hospitalized due to influenza A or B. Significant differences in proportions were present between all groups (P < 0.001). Among ≥18 years old hospitalized participants with influenza, those who had a chest x-ray taken reported a significantly longer length of stay compared to those who had no chest x-ray taken (GM = 2.8; 95% CI 2.7, 3.0 vs GM = 2.5; 95% CI 2.4, 2.6). Those ≥18 years who presented with hypertension as a comorbidity also reported a significantly longer length of stay (GM 3.1; 95% CI 2.6, 3.7) (Table 2).

The associations between sociodemographic factors and clinical signs and symptoms with hospitalization among those <18 years with influenza A or B are presented in Table 3. The model was adjusted for age, gender, ethnicity, fever, cough, chest x-ray. The odds of hospitalization were found to be significantly associated with age, ethnicity, history of fever, cough and chest x-ray taken. Those below 5 compared to 5–17 years were 1.74 times more likely to be hospitalized (adjusted Odds Ratio, aOR 1.74; 95% CI 1.37, 2.48; P < 0.001). The odds of hospitalization were significantly higher for Malays compared to Chinese ethnic groups (aOR 2.32; 95% CI 1.72, 3.12; P < 0.001). The odds of hospitalization were significantly higher for those with a history of fever (aOR 5.58; 95% CI 1.16, 26.88; P = 0.032), cough (aOR 1.75; 95% CI 1.22 2.52; P = 0.002), and those who had a chest x-ray taken at the time of examination (aOR 5.37; 95% CI 3.75, 7.68; P < 0.001). A forest plot showed fever and chest x-ray to have relatively high significance associated to hospitalization in the < 18 years cohort (S1 Fig). The model’s predictive performance, area under the curve (AUC), was reported at 0.717 indicated a good ability to predict hospitalization in those <18 years.

**Table 3:** Associations with hospitalization in Influenza A or B participants < 18 years.

Characteristics	Hospitalization, crude OR (95%CI)	P-value	Hospitalization, adjusted OR (95%CI)	P-value
Age				
< 5 year	1.87 (1.51, 2.31)	<0.001	1.74 (1.39, 2.18)	<0.001
5–17 years	1.00 (reference)		1.00 (reference)	
Gender				
Female	1.00 (reference)		1.00 (reference)	
Male	1.13 (0.92, 1.39)	0.256	1.17 (0.93, 1.49)	0.175
Ethnicity				
Malay	2.64 (2.00, 3.50)	<0.001	2.32 (1.72, 3.12)	<0.001
Chinese	1.00 (reference)		1.00 (reference)	
Indian	1.72 (1.10, 2.70)	0.018	1.50 (0.93, 2.42)	0.096
Others	0.82 (0.51, 1.32)	0.407	0.81 (0.49, 1.33)	0.402
History of:				
Fever	7.58 (1.73, 33.25)	0.007	5.58 (1.16, 26.88)	0.032
Cough	2.15 (1.54, 3.01)	<0.001	1.75 (1.22, 2.52)	0.002
Sore Throat	1.17 (0.94, 1.46)	0.161	–	
Chest X-ray	6.10 (4.30, 8.65)	<0.001	5.37 (3.75, 7.68)	<0.001
Asthma	1.48 (0.83, 2.65)	0.183	–	

The associations between sociodemographic factors, clinical signs and symptoms with hospital duration among those <18 years are presented in Table 4. This model was adjusted for ethnicity, fever, cough, and chest x-ray. Hospital duration ratio was significantly associated with ethnicity, history of fever, cough and those who had a chest x-ray taken. The hospitalization duration ratio was significantly higher for Malays (adjusted Hospitalization Duration Ratio, aHDR 1.22; 95% CI 1.10, 1.34; P < 0.001) and Indians (aHDR 1.32; 95% CI 1.12, 1.52; P = 0.001) compared to Chinese ethnic groups. The hospitalization duration ratio was also significantly higher for those <18 years who had a history of fever (aHDR 1.97; 95% CI 1.05, 3.71; P = 0.034), cough (aHDR 1.14; 95% CI 1.01, 1.28; P = 0.040) and had a chest x-ray taken (aHDR 1.28; 95% CI 1.19, 1.38; P < 0.001). The model diagnostics were reported using model fit statistics including R-squared indicating the model explains approximately 10% of the variance in log transformed hospital duration and F-statistic indicating model was statistically significant overall.

**Table 4:** Associations with hospitalization duration in Influenza A or B participants < 18 years.

	Crude hospitalization duration ratio (95%CI)	P-value	Adjusted hospitalization duration ratio(95%CI)	P-value
Age				
< 5 years	1.03 (0.96, 1.10)	0.419	–	–
5–17 years	1.00 (reference)			
Gender				
Female	1.00 (reference)			
Male	0.99 (0.93, 1.07)	0.860	–	–
Ethnicity				
Malay	1.26 (1.13, 1.39)	<0.001	1.22 (1.10, 1.34)	<0.001
Chinese	1.00 (reference)		1.00 (reference)	
Indian	1.33 (1.14, 1.56)	<0.001	1.32 (1.12, 1.52)	0.001
Others	1.35 (1.12, 1.62)	0.002	1.30 (1.09, 1.56)	0.004
History of:				
Fever	1.87 (0.97, 3.60)	0.061	1.97 (1.05, 3.71)	0.034
Cough	1.21 (1.07, 1.37)	0.003	1.14 (1.01, 1.28)	0.040
Sore Throat	1.03 (0.95, 1.10)	0.504	–	–
Chest X-ray	1.30 (1.21, 1.40)	<0.001	1.28 (1.19, 1.38)	<0.001
Asthma	0.95 (0.80, 1.13)	0.567	–	–

Among participants ≥ 18 years, hospitalization was significantly associated with age, ethnicity, history of fever, cough, sore throat and chest x-ray taken. This model was adjusted for age, ethnicity, fever, cough, sore throat, chest x-ray and hypertension. Those 18–39 years were 1.54 times significantly more likely to be hospitalized (aOR 1.54; 95% CI 1.11, 2.12; P = 0.009) compared to those aged 40–59 years,. The odds of hospitalization were significantly higher for Malays (aOR 1.99; 95% CI 1.43, 2.78; P < 0.001) and Indians (aOR 1.86; 95% CI 1.17, 2.96; P = 0.008) compared to Chinese ethnic groups. The odds of hospitalization were significantly higher for those with a history of fever (aOR 3.10; 95% CI 1.13 8.48; P = 0.028), cough (aOR 1.98; 95% CI 1.22 3.19; P = 0.005), sore throat (aOR 2.07; 95% CI 1.57 2.72; P < 0.001) and those who had a chest x-ray taken (aOR 11.44; 95% CI 7.91, 16.52; P < 0.001) (Table 5). A forest plot showed chest x-ray to have relatively high significance associated to hospitalization in the ≥ 18 years cohort (S2 Fig). The model’s predictive performance, area under the curve (AUC), was reported at 0.797 indicated a good ability to predict hospitalization in adults.

**Table 5:** Associations with hospitalization in Influenza A or B participants aged ≥ 18.

	Hospitalization, crude OR (95%CI)	P-value	Hospitalization, adjusted OR (95%CI)	P-value
Age				
18–39 years	1.17 (0.89, 1.53)	0.254	1.54 (1.11, 2.12)	0.009
40–59 years	1.00 (reference)		1.00 (reference)	
≥ 60 years	1.22 (0.81, 1.83)	0.347	0.83 (0.49, 1.41)	0.493
Gender				
Female	1.00 (reference)			
Male	1.06 (0.84, 1.34)	0.610	–	–
Ethnicity				
Malay	1.86 (1.40, 2.46)	<0.001	1.99 (1.43, 2.78)	<0.001
Chinese	1.00 (reference)		1.00 (reference)	
Indian	1.65 (1.11, 2.46)	0.014	1.86 (1.17, 2.96)	0.008
Others	0.76 (1.46, 1.26)	0.293	0.87 (0.49, 1.55)	0.636
History of:				
Fever	5.23 (2.21, 12.38)	<0.001	3.10 (1.13, 8.48)	0.028
Cough	3.28 (2.13, 5.04)	<0.001	1.98 (1.22, 3.19)	0.005
Sore Throat	2.12 (1.67, 2.69)	<0.001	2.07 (1.57, 2.72)	<0.001
Chest X-ray	10.15 (7.31, 14.10)	<0.001	11.44 (7.91, 16.52)	<0.001
Smoking	2.93 (0.99, 8.62)	0.051	–	–
Asthma	2.27 (1.12, 4.62)	0.023	–	–
Any comorbidity				
Cardiovascular diseases	2.17 (0.36, 13.05)	0.396	–	–
Diabetes mellitus	1.84 (0.94, 3.59)	0.074	–	–
Hypertension	2.20 (1.35, 3.58)	0.002	1.55 (0.82, 2.96)	0.178

The crude and adjusted associations for hospital duration ratio with sociodemographic factors, clinical characteristics and comorbidities among those ≥ 18 years are presented in Table 6. The model was adjusted for age, male, chest x-ray, smoking and hypertension. Hospitalization duration was significantly associated only with age and the need for chest x-ray. Compared to the 40–59 years age group, the hospitalization duration ratio was higher for those in the ≥ 60 years age group (aHDR 1.18; 95% CI 1.01, 1.38; P = 0.032). Compared to females, the percentage of male having a longer duration of hospital stay was 9% lower (aHDR 0.91; 95% CI 0.84, 0.99; P = 0.032). Approximately 11% more of those ≥ 18 years who had taken a chest x-ray reported a significantly longer duration of stay (aOR 1.11; 95% CI 1.01, 1.21; P = 0.028). After adjusting for other covariates, none of the comorbidities examined had any associations with the clinical outcomes.

**Table 6:** Associations with hospitalization duration in Influenza A or B patients ≥ 18 years.

	Crude hospitalization duration ratio (95%CI)	P-value	Adjusted hospitalization duration ratio(95%CI)	P-value
Age				
18–39 years	1.05 (0.95, 1.16)	0.310	1.09 (0.99, 1.20)	0.094
40–59 years	1.00 (reference)		1.00 (reference)	
≥ 60 years	1.25 (1.08, 1.45)	0.003	1.18 (1.01, 1.38)	0.032
Gender				
Female	1.00 (reference)		1.00 (reference)	
Male	0.93 (0.85, 1.01)	0.086	0.91 (0.84, 0.99)	0.032
Ethnicity				
Malay	1.01 (0.91, 1.12)	0.886	–	–
Chinese	1.00 (reference)			
Indian	0.97 (0.83, 1.12)	0.635	–	–
Others	0.98 (0.80, 1.20)	0.842	–	–
History of:				
Fever	0.86 (0.59, 1.26)	0.445	–	–
Cough	1.09 (0.91, 1.30)	0.365	–	–
Sore Throat	0.98 (0.90, 1.07)	0.601	–	–
Chest X-ray	1.13 (1.04, 1.23)	0.004	1.11 (1.01, 1.21)	0.028
Smoking	1.30 (0.97, 1.75)	0.084	1.32 (0.98, 1.78)	0.065
Asthma	1.14 (0.92, 1.42)	0.214	–	–
Any comorbidity				
Cardiovascular diseases	0.68 (0.40, 1.17)	0.167	–	–
Diabetes mellitus	1.22 (0.99, 1.51)	0.064	–	–
Hypercholesterolemia	0.75 (0.30, 1.92)	0.552	–	–
Hypertension	1.18 (1.02, 1.37)	0.028	1.12 (0.95, 1.31)	0.174

## Discussion

Influenza is a significant infectious disease that leads to considerable morbidity and mortality globally each year. A better understanding of the burden and outcomes of influenza infection may increase awareness of the management of the disease. The investigation from this study underscores significant demographic and clinical factors linked to hospitalization and duration for people with influenza. Hospitalization was found to be significantly associated with age, ethnicity, history of fever, cough, and chest x-ray taken for both<18 years and ≥ 18 years. Hospitalization duration was significantly associated with ethnicity, history of fever, cough and chest x-ray for children, and with age and chest x-ray in those ≥ 18 years.

A key finding from this study revealed that those <5 years had a higher likelihood of influenza-related hospitalization compared to those 5–17 years. This finding is consistent with a retrospective review by Li Kim Moy et al. (2017) that examined influenza-related hospitalizations in children aged <16 over three seasons at two major Australian pediatric hospitals [16]. They found that 64% of the 740 total hospitalizations were in children under 5 years old. Similarly, a retrospective study conducted by Low et al. (2022) from 2015–2019 across 12 laboratory sites in Malaysia found that hospitalization rates for influenza peaked in children aged 3–6 years [8].

In Malaysia, the National Immunization Programme (NIP) offers free vaccinations at government clinics to protect children, however, it does not include the influenza vaccine [17]. Limited coverage may reduce its effectiveness as a population-based preventive strategy to reduce the burden of influenza via herd immunity. In addition, caregiver vaccine hesitancy was higher among those with inaccurate beliefs and misconceptions on influenza vaccines [18]. These circumstances further diminish the uptake of vaccinations and may increase adverse health outcomes among children with influenza.

Younger children may be more vulnerable to severe influenza infections due to less developed immune systems [19]. As such, infants and younger children are usually admitted at a significantly lower risk threshold compared to older age groups [14,20,21]. Children may also be more likely to be hospitalized due to delayed symptom recognition [22]. A study on children under five in Peru found that a lack of awareness of signs and symptoms of respiratory tract infections, especially among caregivers or parents with limited education, was a primary factor that led to delays in seeking care [23].

Hospitalization was more likely among younger adults compared to older adults in this study. However, this finding was not consistent with the literature. Plausible factors that may contribute but need further research are the national trends of rising and undiagnosed NCD [24] and delayed medical-seeking behavior. Studies have shown that influenza vaccination rates are often lower in younger adults [25]. They are often less likely to be vaccinated due to low awareness of influenza vaccination, resulting in lower levels of immunity that may require hospitalization once infected with the virus [26].

In both <18 and ≥ 18 years cohorts, Malays and Indians were more likely to be hospitalized and have longer durations of hospitalization compared to Chinese patients. In support of this finding, a study on general admissions in Singapore reported declining hospitalization rates in Chinese compared to Malays and Indians [27]. Furthermore, in Malaysia, the NHMS 2019 report on non-communicable diseases stated that compared to Chinese, Malays have higher rates of hypertension and hypercholesterolemia whereas Indians have higher rates of diabetes and obesity [28]. The pre-existing health conditions among Malays and Indians clearly warrant a higher chance of hospitalization once infected with the influenza virus. The NHMS also reported that risk perception and compliance to the 3 W’s (Wear a mask, Wash your hands, Watch your distance) was higher among the Chinese ethnic but started to wane among the Malays and Indians [28].

The study also revealed that particpants were found more likely to be hospitalized, especially when presented with fever, cough, and coupled with the need for a chest x-ray. The high prevalence of fever and cough suggests that these symptoms were common drivers of the need for inpatient care [29]. The fact that over 80% of hospitalized <18 and ≥ 18 yearsreceived chest x-rays indicates a frequent use of the diagnostic test to assess respiratory complications that require hospitalization. A combination of clinical symptoms and radiographic evaluation can better help to guide the management of influenza in both <18 and ≥ 18 years [30]. The findings from this study show that influenza infections should not be overlooked when fever, cough, and need for a chest x-ray are present. Sore throat was identified as a significant predictive factor for hospitalization only among ≥ 18 years. This is primarily because children may not be able to communicate this information effectively, leading to an inaccurate history of sore throat being reported.

An important finding from this study revealed that compared to the 40–59 years age group, the odds of hospitalization duration were significantly longer for those ≥60 years. This study finding was consistent with previous studies reporting prolonged hospital stays among those aged ≥60 years [6–8,31]. Older people experience more severe illness, potentially due to underlying comorbidities that may exacerbate infections [32,33]. The NHMS 2023 reported that 71% of adults ≥60 years suffer from metabolic syndrome, including elevated blood glucose levels, high blood pressure, raised blood cholesterol, and abdominal obesity [34]. Those ≥60 years often present with a lower prevalence of fever and this may result in misdiagnosis and treatment delays for the elderly population [29,35]. Some elderly may experience immunosenescence, a condition in which both innate and adaptive immune responses are compromised [29,36].

The strengths of this study are that it reflected the patterns of healthcare utilization, health-seeking behavior, and health practices between 2015 and 2019 without being influenced by the changes that took place after the pandemic. Although temporal changes may affect the baseline odds of hospitalization and duration but not the specific associations. This is a postulation but one based on the assumption that the associations are not modified by temporal time. Additionally, during the study period, there were no temporal changes in the population size around the hospital, thus providing population stability. The study also has a few limitations that should be highlighted. Ethnicity is a social construct that combines the effects of one’s genes, environment, social determinants of health, and the complex interplay of these factors. As such, reasons for the differences between ethnicities were undetermined. Additionally, the lack of information on socioeconomic status and healthcare access disparities due to confidentiality from the hospital’s medical records may not address potential confounders. This leads to lack of evidence for causal interpretations due to residual confounding. Also, the study’s focus on a single centre makes the generalizability of the associations to other populations unknown. Apart from these limitations, the introduction of a new respiratory virus screening and identification kit during the study period may increase variation in laboratory testing accuracies.

A few practical recommendations within the Malaysian’s health system may include arranging an annual immunization program as part of school vaccinations for children as well as incorporating vaccination as part of health insurance plan for adults. Additionally, a cost effectiveness study on the inclusion of influenza vaccines in Malaysia’s National Immunization Program (NIP) could benefit these vulnerable populations.

## Conclusion

The findings of this study provide evidence of an association between age, ethnicity, clinical signs, symptoms, and hospitalization and its duration among an urban Malaysian population. Those under 5 years old are more likely to be hospitalized, and those aged 60 and above were more likely to have longer hospital durations compared to all other ages. These age groups may derive higher benefits from primary prevention activities. The heterogeneous associations between the risk factors for hospitalization and hospital duration highlight the need for further research and importance of accounting for crucial socioeconomic, geographic and healthcare access factors. Improved risk classification of people with influenza could potentially lead to more effective treatment strategies for minimizing the consequences of influenza A and B infections.

## Supporting information

S1 Fig Forest Plot of the Odds Ratio of Hospitalization in Influenza A or B participants less than 18 years. (TIF)

S2 Fig Forest Plot of the Odds Ratio of Hospitalization in Influenza A or B participants more than 18 years. (TIF)
