# Supplementary figures and images for: Factors associated with hospitalization of people with influenza in a Malaysian tertiary hospital from 2015 to 2019
Source: PLoS One. 2025 Oct 17;20(10):e0333921. doi: 10.1371/journal.pone.0333921 (PMC12533861; doi:10.1371/journal.pone.0333921)

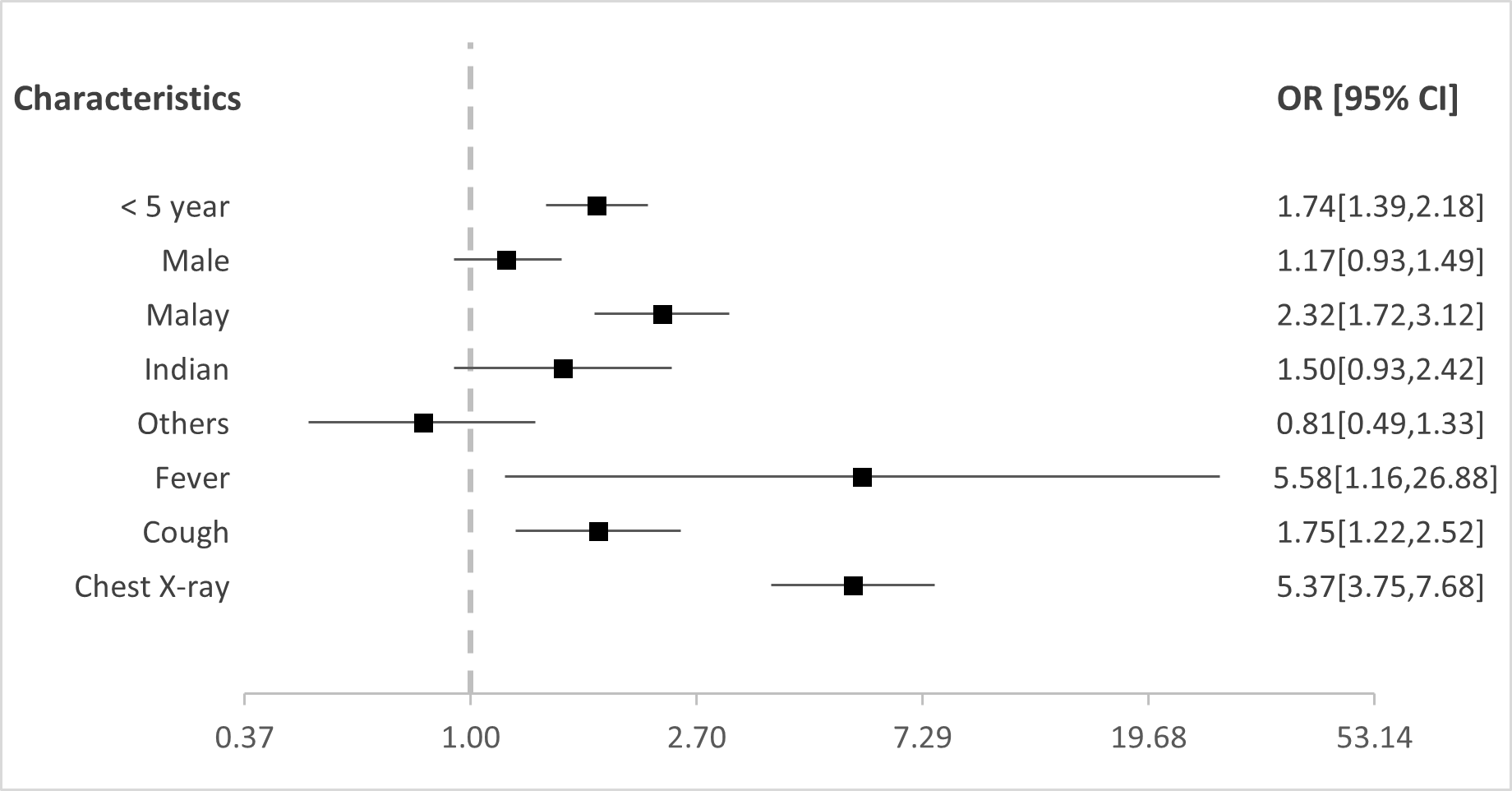

Supplement: S1 Fig — (TIF) [file pone.0333921.s001.tif]

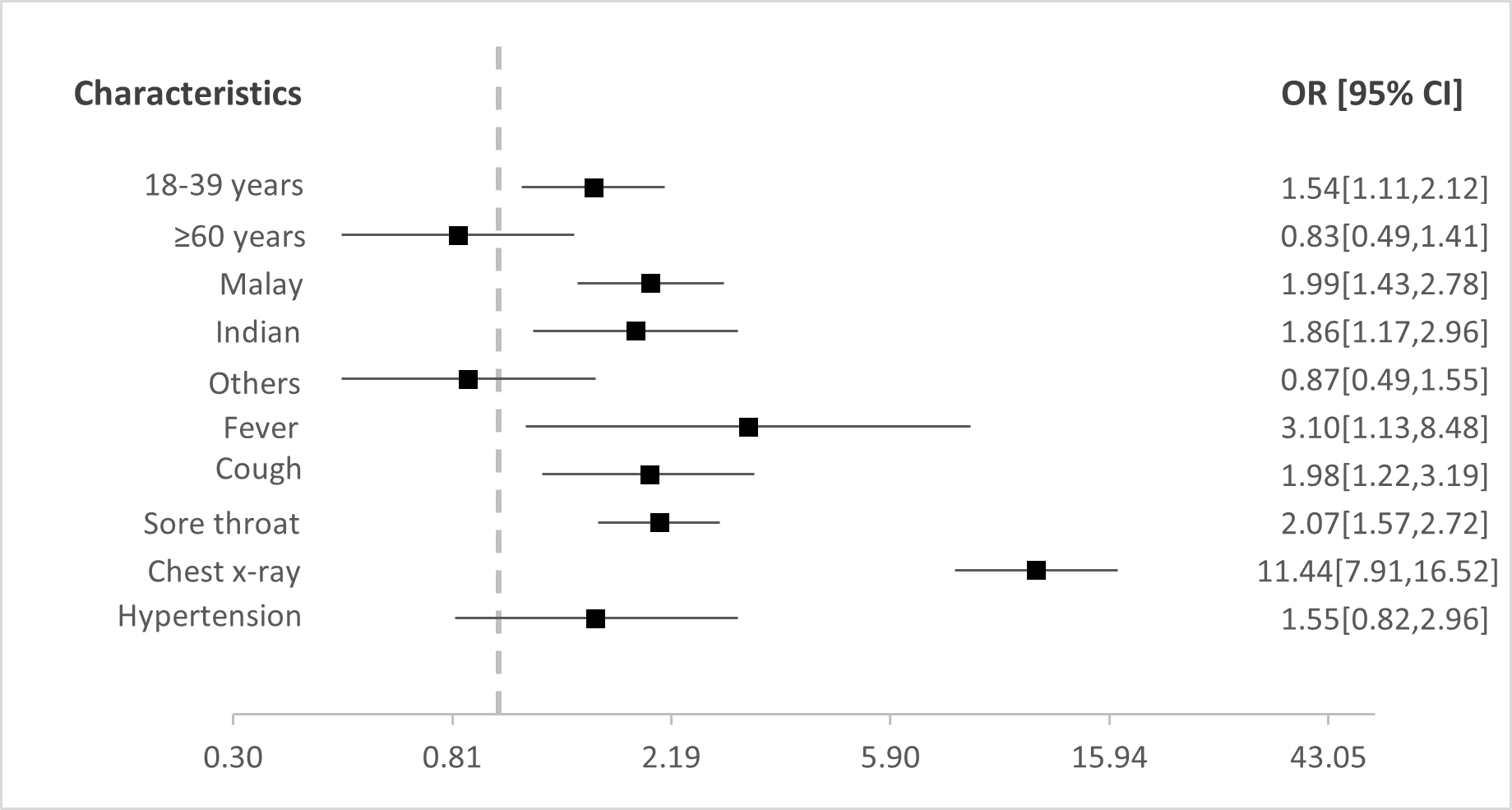

Supplement: S2 Fig — (TIF) [file pone.0333921.s002.tif]
